# Target of rapamycin signaling regulates starch degradation via α-glucan water dikinase in a unicellular red alga

**DOI:** 10.1093/plphys/kiaf106

**Published:** 2025-03-21

**Authors:** Sota Komiya, Imran Pancha, Hiroki Shima, Kazuhiko Igarashi, Kan Tanaka, Sousuke Imamura

**Affiliations:** Laboratory for Chemistry and Life Science, Institute of Integrated Research, Institute of Science Tokyo, 4259-R1 Nagatsutacho, Midori-ku, Yokohama 226-8503, Japan; Laboratory for Chemistry and Life Science, Institute of Integrated Research, Institute of Science Tokyo, 4259-R1 Nagatsutacho, Midori-ku, Yokohama 226-8503, Japan; Department of Industrial Biotechnology, Gujarat Biotechnology University, Gandhinagar, Gujarat 382355, India; Department of Biochemistry, Tohoku University Graduate School of Medicine, Seiryo-machi 2-1, Aoba-ku, Sendai 980-8575, Japan; Department of Biochemistry, Tohoku University Graduate School of Medicine, Seiryo-machi 2-1, Aoba-ku, Sendai 980-8575, Japan; Laboratory for Chemistry and Life Science, Institute of Integrated Research, Institute of Science Tokyo, 4259-R1 Nagatsutacho, Midori-ku, Yokohama 226-8503, Japan; Laboratory for Chemistry and Life Science, Institute of Integrated Research, Institute of Science Tokyo, 4259-R1 Nagatsutacho, Midori-ku, Yokohama 226-8503, Japan; Space Environment and Energy Laboratories, NTT Corporation, Musashino-shi, Tokyo 180-8585, Japan

## Abstract

Target of rapamycin (TOR) signaling pathways are major regulators of starch accumulation in various eukaryotes. However, the underlying molecular mechanisms of this regulation remain elusive. Here, we report the role of TOR signaling in starch degradation in the unicellular red alga *Cyanidioschyzon merolae*. Reanalysis of our previously published phosphoproteome data showed that phosphorylation of the serine residue at position 264 of a protein similar to α-glucan water dikinase (CmGWD), a key regulator of starch degradation, was not increased by rapamycin treatment. In the CmGWD knockout strain, starch content increased and starch phosphorylation decreased, indicating that CmGWD is a functional GWD. CmGWD-dependent starch degradation under dark conditions was alleviated by rapamycin treatment. The overexpression of a phosphomimic CmGWD variant, in which Ser264 was replaced by aspartic acid, or a dephosphomimic CmGWD variant, in which Ser264 was replaced by alanine, resulted in 0.6-fold lower and 1.6-fold higher starch accumulation compared to the wild-type CmGWD-overexpressing strain, respectively. The starch levels corresponded with starch phosphorylation status. Furthermore, the dephosphomimic CmGWD-overexpressing strain accumulated nearly the same amount of starch with or without rapamycin treatment as the rapamycin-treated wild-type CmGWD-overexpressing strain. In contrast, rapamycin treatment did not trigger an increase in starch accumulation in the phosphomimic CmGWD-overexpressing strain. These results indicate that TOR signaling regulates starch degradation in *C. merolae* by altering the phosphorylation state of Ser264 in CmGWD.

## Teaser

Target of rapamycin signaling enhances starch contents by regulating the activity of α-glucan water dikinase, through changing its phosphorylation status in the red alga *Cyanidioschyzon merolae*.

## Introduction

Biomass is attracting attention as an alternative bioenergy resource to fossil fuels. In particular, biomass generated using microalgae is considered as a useful renewable energy resource because its biomass productivity per unit time and unit area is higher than that of other photosynthetic organisms such as land plants, and it does not compete with resources used for food production (Chisti 2007). Microalgae fix atmospheric CO_2_ by photosynthesis and convert them into energy reserve compounds such as triacylglycerols (TAGs) and starch. The TAGs, a compound consisting of 1 molecule of glycerol bound to 3 molecules of fatty acids, can be used to produce liquid fuels such as biodiesel (Chisti 2007). Starch, on the other hand, is a polymeric compound consisting of a large amount of α-glucose polymerized by glycosidic bonds and is used as a raw material for producing liquid fuels such as bioethanol (John et al. 2011). A recent study has also shown that the starch remaining after TAG extraction in algal biomass can be converted into methyl levulinic acid, a raw material used in the pharmaceutical and cosmetics industries, and methyl lactate, a raw material for preparing biodegradable plastics using chemical catalysts (Yamaguchi et al. 2017). These compounds have higher value than biofuels, and starch produced by algae is a good feedstock that can be converted to industrially relevant products unlike TAG. Therefore, an increase in the starch content will lead to an increase in the overall value of the algal biomass.

Microalgae, similar to land plants, perform photosynthesis during the day and accumulate the starch, during the night break down the accumulated starch to obtain energy for metabolism and growth (Smith et al. 2005; Zeeman et al. 2007). Apart from the day/night cycle, it is generally known that microalgae accumulate starch under various stress conditions such as nutrient starvation like nitrogen starvation (Pancha et al. 2014; Pancha et al. 2019). These indicate that photosynthetic organisms contain different mechanisms for synthesis and degradation of starch. Several mechanisms have been reported to regulate starch levels. Examples include regulation by T6P, allosteric regulation of ADP-glucose pyrophosphorylase (AGPase), circadian rhythms, temperature, redox, and the target of rapamycin (TOR) (Geigenberger 2011; Caldana et al. 2013; Jüppner et al. 2018; Pancha et al. 2019). The first example that is highly relevant to this study is the regulation of starch synthesis through redox mechanisms, which involve chloroplast thioredoxins (Trx) and NADP-thioredoxin reductase C (Skryhan et al. 2018). Many enzymes involved in carbohydrate metabolism such as AGPase, BAM1, and AMY3 are redox-dependent regulated. In vitro studies have verified redox regulation activities of these enzymes dependent on physiological reductants and protein cysteine residues (Skryhan et al. 2018).

The second example relevant to this study is the regulation of starch synthesis by TOR (Caldana et al. 2013; Jüppner et al. 2018; Pancha et al. 2019). TOR is a serine/threonine kinase conserved in all eukaryotes and plays a key role in various cellular processes such as cell growth, metabolism, translation, etc. (Laplante and Sabatini 2012). In yeast and mammals, TOR exists in 2 structurally and functionally distinct protein complexes, known as TOR complex 1 and TOR complex 2, whereas in plant lineages, only TOR complex 1 has been identified so far (Heitman et al. 1991). TOR complex 1 is involved in cell growth and metabolism in response to nutritional status and energy availability, and its activity is specifically inhibited by rapamycin (Heitman et al. 1991). Previous studies using algae and plants have indicated that inhibiting TOR with rapamycin increases starch accumulation in the cell (Jüppner et al. 2018; Pancha et al. 2019; Han et al. 2022). Recently, our research group using the unicellular red alga *Cyanidioschyzon merolae* identified a mechanism through which the TOR signaling pathway regulates starch synthesis (Pancha et al. 2019). The serine-613 residue of CmGLG1, which is homologous to glycogenin, an enzyme that initiates the starch/glycogen synthesis in yeast and mammals, is phosphorylated by the TOR signaling pathway, and its phosphorylation status regulates starch accumulation in *C. merolae* (Pancha et al. 2019).

On the other hand, the starch degradation mechanism has been elucidated using model plant *Arabidopsis thaliana* (Silver et al. 2014). During starch degradation, the first step is the phosphorylation of the surface of the starch granules to degrade the outer semicrystalline structure of the starch. As a first step in the process, glucan water dikinase (GWD) and phosphoglucan water dikinase (PWD) phosphorylate C6 and C3 position of the glucose in starch, respectively (Ritte et al. 2006). Phosphorylation of C6 was done by GWD is responsible for initiating hydration of the granule surface (Ritte et al. 2006), while phosphorylation of C3 by PWD is responsible for inducing steric distortion that prevents helical disruption and recrystallization (Hejazi et al. 2009). Further, the exposure of the glucan chain is a key step in the starch degradation process. Once the glucan chain is exposed, α-1,4 glycosidic bonds are subsequently cleaved by β-amylase 1 (BAM1) and β-amylase 3 (BAM3) (Fulton et al. 2008). Next, the glucan phosphatases, STARCH EXCESS4 (SEX4) and LSF2, dephosphorylate glucose phosphorylated by GWD and PWD further promoting hydrolysis by BAM1 and BAM3 (Santelia et al. 2011). Apart from these enzymes, various other enzymes are also involved in starch degradation process such as LSF1, a protein that serves as a scaffold to bind with BAM3 and BAM1 (Schreier et al. 2019). Cleavage of the α-1,6-branch is also carried out by isoamylase (ISA3) and limit dextrinase (LDA) (Streb and Zeeman 2012). The α-amylase (AMY3) is also thought to be involved in degrading starch on the granule surface (Seung et al. 2013). With the involvement of various enzymes, starch is finally converted into maltose and glucose. Similar to starch synthesis, its degradation also occurs in chloroplast, and many enzymes involved in the process are regulated through redox regulation (Santelia et al. 2015). However, there are still many unanswered questions regarding the regulatory mechanisms of starch degradation that need to be clarified.

*C. merolae* used in this study is a unicellular red alga that lives in hot and acidic environments (pH 1–3, 40–50 °C) (Matsuzaki et al. 2004). The *C. merolae* contain relatively small genome size (16.5 Mb) and low genetic redundancy; additionally, all the 3 organelles' genomes are fully sequenced and publicly available (Matsuzaki et al. 2004; Nozaki et al. 2007). Furthermore, various genetic, molecular biological, and biochemical tools have been used that establish *C. merolae* as a model system for photosynthetic eukaryotes (Imamura et al. 2008; Ohnuma et al. 2008; Miyagishima and Tanaka 2021). The red alga *C. merolae* accumulate floridean starch in the cytoplasm of the cells not in the chloroplast-like green algae. Furthermore, the floridean starch (hereafter, starch) mainly contains semi-amylopectin-type glucan (Hirabaru et al. 2010). The aim of the present study was to elucidate the regulatory mechanism of starch degradation using *C. merolae*. To this end, we reanalyzed our previously published phosphoproteome data under TOR inactivation using rapamycin in *C. merolae* (Pancha et al. 2019). Our reanalysis indicates that GWD is possibly regulated through phosphorylation by the TOR signaling pathway. Further detailed analysis revealed that TOR regulates starch degradation in *C. merolae* by altering the phosphorylation status of the CmGWD Ser264 residue.

## Results

### Phosphorylation of CmGWD is regulated by the TOR signaling pathway

**Figure 1.**
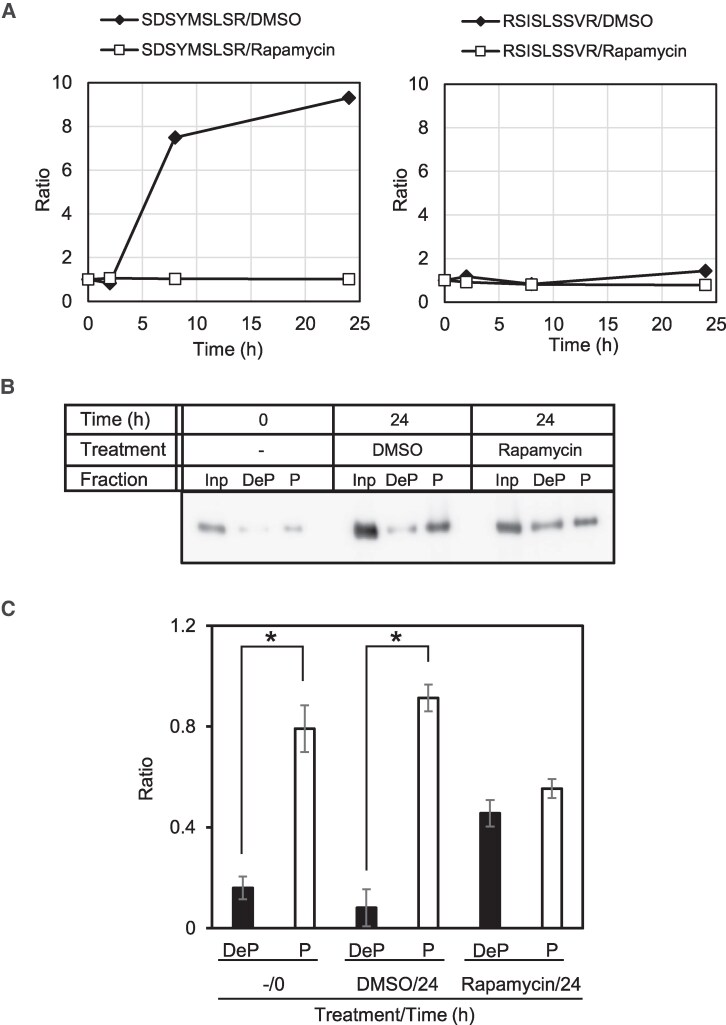
TOR-dependent phosphorylation of CmGWD. **A)** Phosphorylation pattern of CmGWD under rapamycin treatment detected with LC-MS/MS analysis. The changes are shown in the amount of the mono-phosphorylated 262-SDSYMSLSR-270 (left) and 288-RSISLSSVR-296 (right) peptides of the CmGWD protein in the *C. merolae* SF12 strain treated with 2 *µ*M rapamycin or the solvent DMSO alone, which was examined by quantitative LC-MS/MS using iTRAQ in a previous study (Pancha et al. 2019). The LC-MS/MS analysis was conducted on a single sample (*n* = 1). However, by performing temporal sampling and analysis, accurate phosphorylation patterns were obtained. **B)** Phosphorylation status of CmGWD under rapamycin treatment conditions as determined by the Phos-tag agarose analysis. Aliquot of 15 *µ*g of protein obtained from the Flag-fused CmGWD expressing strain was loaded onto Phos-tag agarose. The dephosphorylated fraction (DeP) and phosphorylated fraction (P) were collected as described in the Experimental procedures section. Both fractions along with 15 *µ*g total protein as an input (Inp) were determined by immunoblotting with anti-FLAG antibodies. Other details are as described in **A)**. **C)** Ratio of the phosphorylation status of CmGWD under rapamycin treatment conditions as determined by the Phos-tag agarose analysis. After detecting FLAG-fused CmGWD in the 3 fractions, the signal intensities of DeP and P were divided by those of Inp to obtain the ratio of phosphorylated protein/total protein. The values are averages of 3 independent experiments. Error bars and asterisks indicate the standard deviation and significant difference between 2 treatments (Student's *t*-test, *P* < 0.05), respectively.

In our previously published phosphoproteomic analysis using liquid chromatography-tandem mass spectrometry (LC-MS/MS), we showed that the *C. merolae* TOR signaling pathway regulates starch synthesis via phosphorylation of CmGLG1 (Pancha et al. 2019). During that study, we only considered the proteins as potential TOR substrates whose phosphorylation was reduced after TOR inactivation by rapamycin treatment among proteins related to starch synthesis and degradation (Pancha et al. 2019). However, the proteins whose phosphorylation was increased in the control condition (DMSO treatment) but not increased by rapamycin treatment were not considered (Pancha et al. 2019). In this study, we rechecked those excluded proteins and found a protein CMT547C (gene number in the *C. merolae* database) (http://czon.jp), which is annotated as “starch associated protein R1, alpha-glucan water dikinase” (hereafter, CmGWD), as the candidate protein for this study. The previous quantitative LC-MS/MS using the iTRAQ isobaric mass tags identified and quantified 2 mono-phosphorylated peptides of CmGWD: 262-SDSYMSLSR-270 and 288-RSISLSSVR-296 (Supplementary Fig. S1). Figure 1A shows the change in the quantity of the phosphorylated 262-270 and 288-296 peptides of CmGWD obtained by the relative intensity values of the iTRAQ reporter ions. Phosphorylated 262-270 was increased in the control (DMSO treatment), whereas no increase was observed with rapamycin treatment. The phosphorylated 288-296 peptide did not show such a remarkable change. These results suggested that the TOR signaling pathway regulates at least some of the phosphorylations on CmGWD. To further support the hypothesis, phosphorylation status of CmGWD was also determined using Phos-tag agarose (Kinoshita et al. 2005; Pancha et al. 2019) (Fig. 1B and 1C). In this analysis, the agarose captures phosphorylated proteins, while dephosphorylated proteins pass through the agarose and are collected as the flow-through fraction. For this purpose, a strain expressing CmGWD with a FLAG-tag fused to the C-terminus was constructed, and the protein was extracted 24 h after rapamycin or DMSO treatment, and phosphorylation of CmGWD was analyzed using Phos-tag agarose followed by immunoblot analysis. The results indicated that ratios of phosphorylated and dephosphorylated CmGWD at 0 and 24 h after DMSO treatment are similar. However, the phosphorylation of CmGWD in rapamycin-treated cells at 24 h was clearly less efficient compared to that in DMSO-treated cells (Fig. 1B), and the amount of phosphorylated and dephosphorylated CmGWD was almost the same (Fig. 1C). It is noteworthy that the levels of FLAG-fused CmGWD were almost the same irrespective of rapamycin treatment (Supplementary Fig. S2). These results indicate that phosphorylation status of CmGWD is regulated through TOR signaling in *C. merolae.*

### CmGWD is involved in starch phosphorylation and degradation

**Figure 2.**
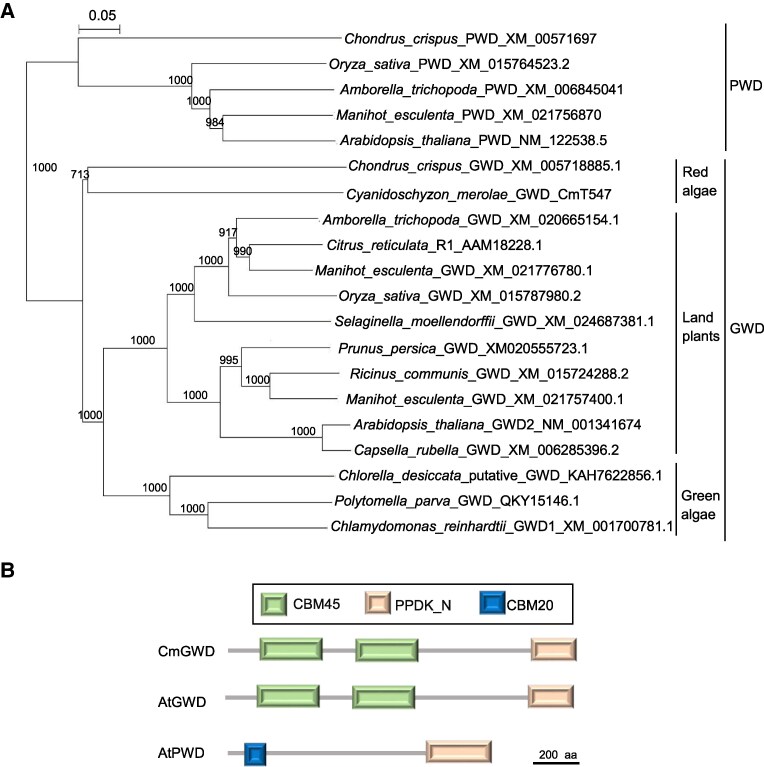
Phylogenetic tree of algal and plant GWDs and PWDs and domain structure of CmGWD. **A)** Phylogenetic tree of algal and plant GWDs and PWDs. A neighbor joining tree was constructed on the basis of 927 unambiguously aligned amino acid proteins. Numbers at each node represent percentages from 1,000 bootstrap replications. Branch lengths are proportional to the number of amino acid substitutions, as indicated by the scale bar on top of the tree. **B)** Comparison of domain structures of CmGWD, AtGWD, and AtPWD. All domains were predicted using SMART algorithms. Both GWD and PWD contain C-terminal PPDK_N domain while GWDs and PWD contain CBM45 module and CBM20 module at their N-terminal, respectively.

As there is no report available on the function of GWD in red algae, we first analyzed phylogenetic and structural analysis of CmGWD before dissecting the relationship between CmGWD and TOR signaling. Phylogenetic analysis using amino acid sequences of GWD and PWD from green algae and land plants along with CmGWD revealed that the phylogenetic tree can be mainly divided into 2 groups: GWD and PWD (Fig. 2A). CmGWD is clearly clustered with GWD of green algae and land plants together with red algal GWD (Fig. 2A). Further, we also compared the secondary structure of CmGWD with *A. thaliana* GWD and PWD (Fig. 2B). The results indicate that CmGWD, like *A. thaliana* GWD, contained CBM45 (carbohydrate-binding module 45) and PPDK_N (pyruvate phosphate dikinase) domains at the N- and C-terminal, respectively. *A. thaliana* PWD has a CBM20 at the N-terminus in addition to PPDK_N, showing a domain structure different from both GWDs.

**Figure 3.**
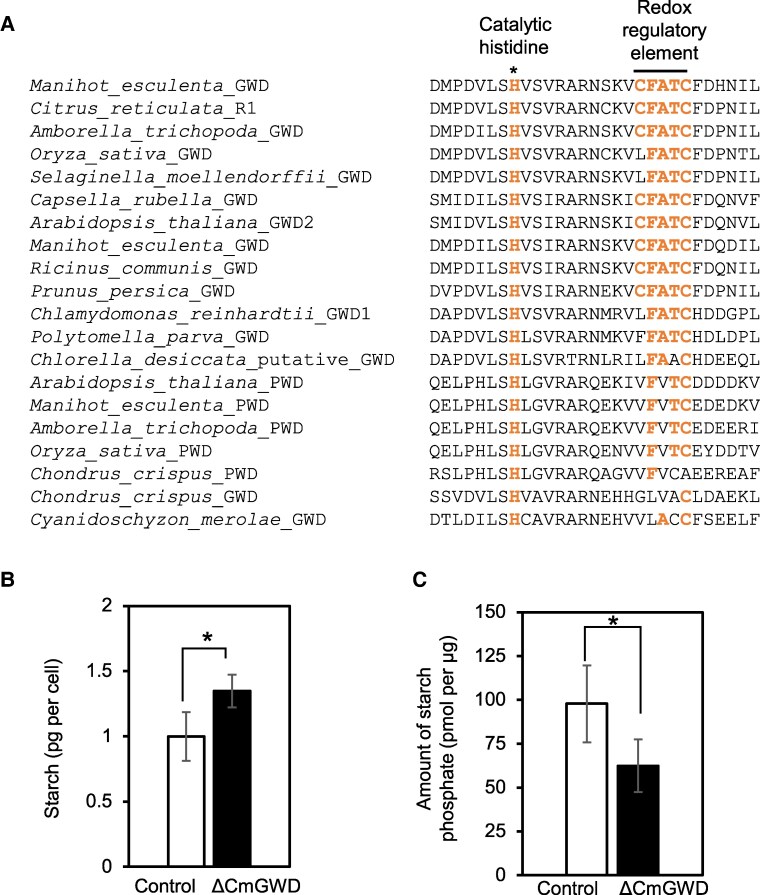
Regulation and function of CmGWD. **A)** Partial amino acid sequence alignment of GWDs and PWDs. Each accession number is shown after each name of an organism and protein in Fig. 2A. Asterisk and bar indicate conserved catalytic histidine residue and regulatory CFATC domain (redox regulatory element), respectively. The bold characters indicates the conserved amino acids. **B and C)** CmGWD regulates the amounts of starch and starch phosphate. *C. merolae* SF12 and ΔCmGWD cells were grown under normal conditions until OD_750_ = 0.2 to 0.4. The cell number, starch content, and amount of starch phosphate were analyzed. The starch content is presented in pg per cell (B) while starch phosphate is presented in pmol per µg (C). The values are averages of 3 independent experiments, error bars indicate standard deviation, and asterisks indicate a significant difference between 2 treatments (Student's *t*-test, *P* < 0.05).

An amino acid alignment analysis also indicated that the histidine residue, which is responsible for the catalysis of GWD, is completely conserved among proteins in land plants and green algae (Fig. 3A). However, the CFATC domain, which is responsible for redox regulation of GWD, was conserved in many land plants (Mikkelsen et al. 2004, 2005; Mdodana et al. 2019) but not in green algae and red algae including *Chondrus crispus* and *C. merolae* (Fig. 3A). This implies that the redox regulation of GWD is not conserved in red algae, and the other mechanism(s) such as phosphorylation status of GWD might regulate its activity.

Next, to clarify the function of CmGWD genetically, we constructed a CmGWD knockout strain (Supplementary Fig. S3). The resulting CmGWD knockout strain and its parental strain, SF12, were assayed for the amount of starch accumulation. The results showed that starch accumulation in the CmGWD knockout strain was significantly higher (1.4-fold) than that in the SF12 strain (Fig. 3B). We also investigated the phosphorylation of the extracted starch from both strains. The amount of phosphorylation per starch in the CmGWD knockout strain was significantly lower (0.6-fold) than that in the control strain, SF12 (Fig. 3C). These results indicate that CmGWD is involved in degrading starch by enhancing starch's phosphorylation, like GWD in other organisms.

### Starch degradation by CmGWD during dark conditions is regulated by TOR signaling

**Figure 4.**
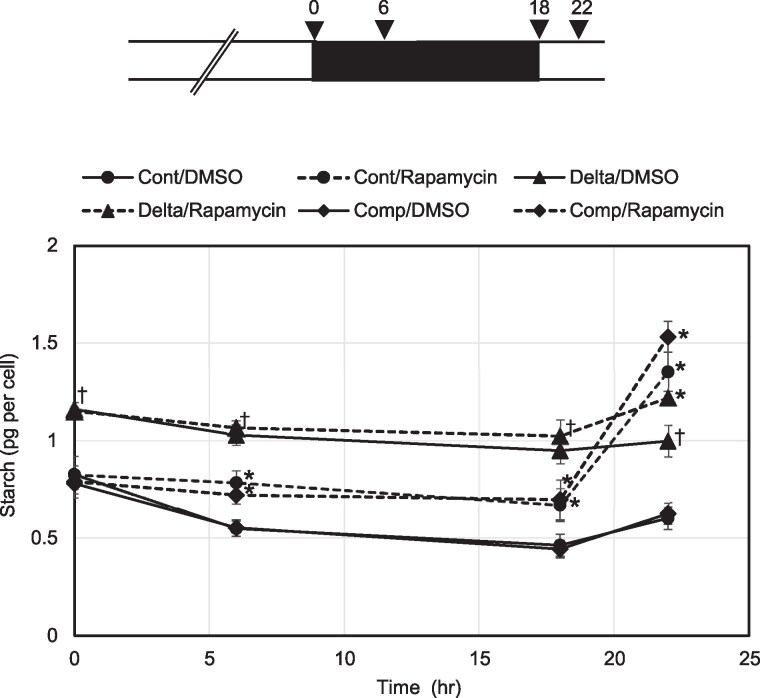
Starch degradation by CmGWD during dark conditions is regulated by TOR signaling. *C. merolae* SF12 (Cont), ΔCmGWD (Delta), and CmGWD complementation in the CmGWD knockout (Comp) were grown under normal conditions until OD_750_ = 0.3 to 0.4. The light for the cultures was turned off for 18 h and then turned on again as shown at the top. Time 0 was set when the light was turned off. Rapamycin and its solvent, DMSO, were added to the culture at time 0. The cell number and starch content were analyzed at 0, 6, 18, and 22 h. The starch content is presented in pg per cell at the bottom. The values are averages of 3 independent experiments, and error bars indicate standard deviations. * indicates a significant difference between DMSO and rapamycin treatment at each time point in the same strains. ^†^ indicates a significant difference between Comp or Delta and Cont under DMSO treatment at each time point.

In photosynthetic organisms, starch is synthesized during the daytime, and starch levels decrease during dark conditions (Zeeman et al. 2007). We thus examined whether CmGWD is involved in the dark-induced starch degradation using the CmGWD knockout strain (Fig. 4). As in other organisms, starch levels decreased and increased during dark and light conditions, respectively, in the WT strain. In the CmGWD knockout strain, starch levels reduced; however, the reduction ratios before the dark condition were significantly relieved compared to those of the WT strain: WT, 0.66 and 0.56; CmGWD-knockout strain, 0.88 and 0.82 (ratio at dark 6 h/0 h and dark 18 h/0 h). Note that the basal starch levels in the knockout strain were higher than WT, as indicated in Fig. 3B. The pattern of fluctuation of starch was almost the same as that of WT in the CmGWD complementation in the CmGWD knockout strain (Fig. 4; Supplementary Fig. S4). These results indicate that CmGWD is involved in starch degradation during dark conditions.

To check whether the CmGWD-dependent starch degradation is regulated by TOR signaling, we added rapamycin to the cultures and checked the starch levels. As shown in Fig. 4, starch barely decreased during dark conditions in WT and the complement strains. However, the patterns of starch levels were not significantly changed in the CmGWD knockout strain with rapamycin treatment. These results indicate that CmGWD function is controlled by TOR signaling. Interestingly, starch levels in rapamycin-treated WT, and the complement strains were clearly induced after light exposure. This induction was observed in the CmGWD knockout strain, but to a lesser degree than in the other 2 strains. These results strongly support our hypothesis that TOR signaling regulates starch levels by altering CmGWD function.

### Phosphorylation status of serine 264 residue of CmGWD is crucial for starch accumulation

**Figure 5.**
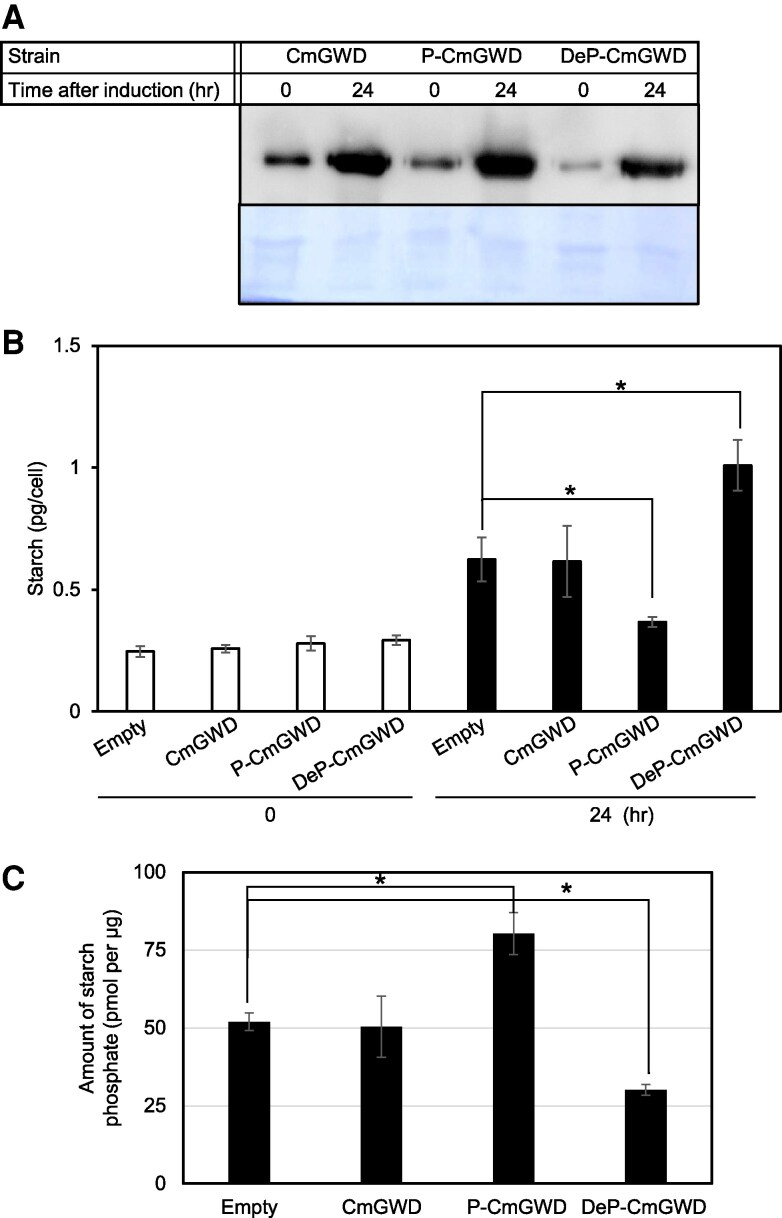
Dephosphorylation status of serine 264 residue of CmGWD is crucial for starch accumulation. **A)** CmGWD expression level analysis in nitrate inducible expression system by immunoblot analysis. Wild-type CmGWDox (CmGWD), CmGWD^S264A^ox (DeP-CmGWD), and CmGWD^S264D^ox (P-CmGWD) cells were first grown in ammonium containing medium until OD_750_ = 0.5 to 0.7, and then, cells were collected by centrifugation and gently resuspended in a nitrate-containing medium. After 24 h of growth in the nitrate-containing medium, the total protein was extracted, and the abundances of CmGWD, CmGWD^S264A^, and CmGWD^S264D^ proteins were analyzed by immunoblotting analysis using anti-FLAG antibodies. After antibody detection of the signal, the membrane was stained with Coomassie Brilliant Blue, which was used as a loading control (lower panel). **B)** Role of phosphorylation of CmGWD Ser264 in starch accumulation. For starch content analysis, after transferring the cells to the nitrate-containing medium, cell number and starch content were analyzed. The starch content is presented in pg per cell. The time on the *x*-axis represents the duration after the cells were transferred to the nitrate-containing medium. The values are averages of 3 independent experiments, error bars indicate standard deviation, and asterisks indicate a significant difference between the treatments (Student's *t*-test, *P* < 0.05). **C)** Starch phosphate levels after induction of each CmGWD protein. The cell number, starch content, and amount of starch phosphate were analyzed 24 h after transfer to the nitrate-containing growth medium in the indicated strains. The values are averages of 3 independent experiments, error bars indicate standard deviations, and asterisks indicate a significant difference between 2 treatments (Student's *t*-test, *P* < 0.05).

Next, we revealed the relationship between the phosphorylation status of CmGWD and starch accumulation in the cell. To analyze this, we constructed a FLAG-fused phosphomimic and FLAG-fused dephosphomimic CmGWD-overexpressing strains. Since the LC-MS/MS result of phosphopeptide 262-270 had identified Ser264 as the most probable phosphorylation site (Supplementary Fig. S1), we replaced Ser264 with aspartic acid or alanine to mimic phosphorylated Ser264 (P-CmGWD) or dephosphorylated Ser264 (DeP-CmGWD), respectively. To evaluate the effect of the changes on starch accumulation, a wild-type CmGWD (CmGWD) expressing strain was also constructed. The expressions of these phosphomimic and dephosphomimic CmGWDs were controlled by the promoter of the nitrite reductase gene, and the protein levels in these strains can be induced by changing the nitrogen source in the growth medium from ammonia to nitrate (Imamura et al. 2010; 2018). As the control, an empty vector transformed strain (Empty) was also constructed. Expression levels of each FLAG-fused CmGWD protein were analyzed by immunoblot analysis using anti-FLAG antibodies to check whether each protein was increased after changing the nitrogen source in the growth medium from ammonia to nitrate. The results showed that the expression levels of all types of CmGWD were clearly higher in cells grown in the nitrate condition than in those grown in the ammonium condition (Fig. 5A).

Further, we also quantified the amount of starch in each strain at 24 h after transfer to the nitrate-containing growth medium and compared it with that at 0 h (ammonia growth condition). The results indicate that approximately 2.5-fold higher starch and 2.4-fold higher starch were accumulated in the control and wild-type CmGWD-overexpressing strains compared with that at 0 h, respectively (Fig. 5B). It is here noted that ammonia is the preferred nitrogen source for the *C. merolae* cells, so transferring cells from ammonia to the nitrate-containing medium might result in a nitrogen depletion-like response in *C. merolae* cells (Imamura et al. 2009; 2010). Surprisingly, the phosphomimic CmGWD overexpression strain showed lower starch accumulation, about 0.6-fold, than the wild-type CmGWD-overexpressing strain, while the dephosphomimic CmGWD-overexpressing strain showed a 3.5-fold increase (Fig. 5B). Under the same conditions, 24 h after transfer to the nitrate-containing growth medium, we also investigated the phosphorylation of the extracted starch from the 4 strains. As shown in Fig. 5C, the amount of phosphorylation per starch in the phosphomimic CmGWD and dephosphomimic CmGWD overexpression strains was significantly higher (1.5-fold) and lower (0.6-fold) than that in the control strain, respectively. These results indicate that phosphorylated Ser264 of CmGWD decreases the starch accumulation and dephosphorylated Ser264 of CmGWD increases starch accumulation by changing the starch phosphorylation levels in the cell.

### Phosphorylation status of Ser264 of CmGWD is regulated by TOR signaling pathway

**Figure 6.**
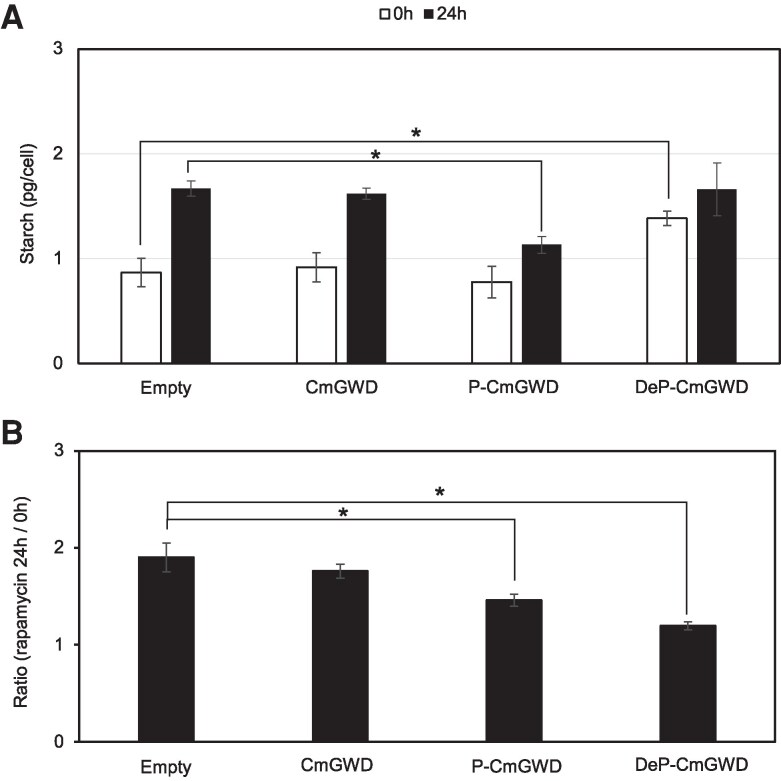
The role of phosphorylation status of Ser264 of CmGWD in starch accumulation is regulated by TOR signaling pathway. **A)** Role of phosphorylation of CmGWD Ser264 by TOR signaling in starch accumulation. CmGWDox (CmGWD), CmGWD^S264A^ox (DeP-CmGWD), and CmGWD^S264D^ox (P-CmGWD) were grown under TOR inactivation conditions by rapamycin. Empty represents a control strain of CmGWD, P-CmGWD, and DeP-CmGWD. After 24 h rapamycin treatment, starch contents in each strain were measured the same as in Fig. 3B. The starch content is presented in pg per cell. The values are averages of 3 independent experiments, error bars indicate standard deviation, and asterisks indicate a significant difference between 2 treatments (Student's *t*-test, *P* < 0.05). **B)** Ratio of the starch amounts before and after rapamycin treatment. The induction ratio was calculated by dividing the amount of starch 24 h after rapamycin treatment measured in panel A by the amount of starch at 0 h. The values are averages of 3 independent experiments, error bars indicate standard deviation, and asterisks indicate a significant difference between 2 treatments (Student's *t*-test, *P* < 0.05).

To determine the relationship between the TOR signaling pathway and phosphorylation status of Ser264 of CmGWD, we constructed strains in which CmGWD, P-CmGWD, or DeP-CmGWD is constitutively overexpressing, and the starch contents were measured at 0 and 24 h after rapamycin treatment (Fig. 6A). As the control, an empty vector transformed strain (Empty) was also constructed and analyzed. The results showed that an increase in starch accumulation after rapamycin treatment was observed in the control strain as previously reported (Pancha et al. 2019). Almost the same amount of increase in starch content was also observed in the wild-type CmGWD-overexpressing strain after rapamycin treatment. In contrast, the phosphomimic CmGWD overexpression strain showed a significant (0.68-fold) decrease in starch compared with the control strain. In the case of dephosphomimic CmGWD-overexpressing strain, no significant difference in starch content was observed compared with the control strain. Furthermore, no significant difference in starch amount at 0 and 24 h was observed in the dephosphomimic CmGWD-overexpressing strain after rapamycin treatment (Fig. 6B). Also, the starch amount at 0 h in the dephosphomimic CmGWD-overexpressing strain was almost the same as that in the rapamycin-treated control strain (Fig. 6A). It is noteworthy that the levels of FLAG-fused CmGWD, phosphomimic CmGWD, and dephosphomimic CmGWD were almost the same irrespective of rapamycin treatment (Supplementary Fig. S2). These results indicate that the phosphorylation of Ser264 is regulated by the TOR signaling pathway and also support our hypothesis that phosphorylation status of Ser264 is crucial for starch accumulation in *C. merolae*.

## Discussion

**Figure 7.**
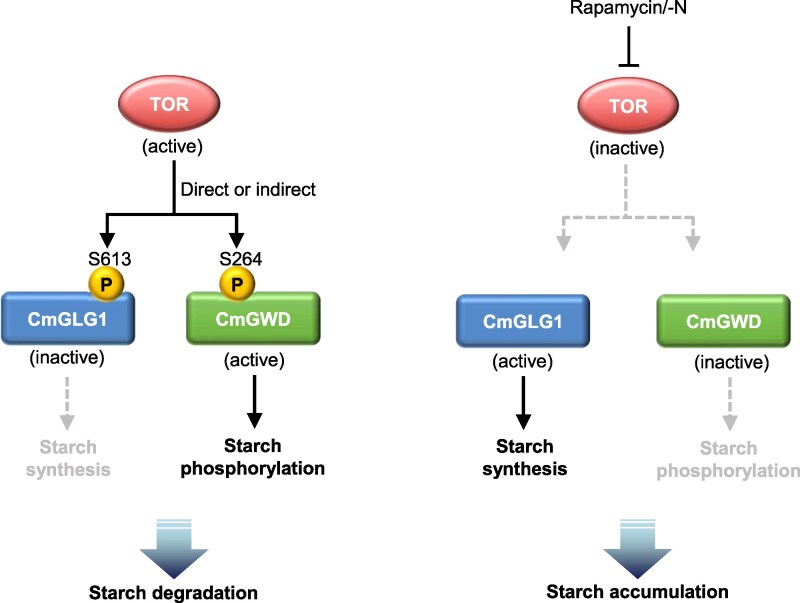
Possible model for the starch accumulation based on phosphorylation status of CmGLG1 and CmGWD in response to target of rapamycin (TOR) signaling. The left and right panels of the figure represent potential models of starch degradation and accumulation, respectively. The “P” in the background of the yellow circles denotes the phosphorylation of the specified amino acid residues. Solid lines indicate that the respective proteins are functional, while gray dashed lines indicate that the function is absent or reduced. When starch is degraded, CmGWD and CmGLG1 are phosphorylated directly or indirectly by the TOR signaling pathway. Consequently, CmGWD becomes activated and phosphorylates starch, whereas CmGLG1 becomes inactivated, resulting in a reduction in de novo starch synthesis and the promotion of starch degradation. Conversely, when TOR is inactivated (e.g. by rapamycin treatment or nitrogen deficiency [-N]), phosphorylation of CmGWD and CmGLG1 does not occur. In this case, CmGWD becomes inactivated, and CmGLG1 is activated. This leads to a decrease in starch phosphorylation and an increase in de novo starch synthesis, thereby promoting starch accumulation.

Starch amount fluctuates under some physiological conditions, such as the day/night cycle, and accumulates under stress conditions such as nitrogen depletion in the land plants and algae (Geigenberger 2011; Caldana et al. 2013; Pancha et al. 2014; Choi et al. 2018; Jüppner et al. 2018; Pancha et al. 2019). Furthermore, the findings of various research groups have revealed that starch is also accumulated by TOR inactivation (Caldana et al. 2013; Jüppner et al. 2018; Pancha et al. 2019). Additionally, our previous study revealed that starch synthesis is enhanced by dephosphorylation of CmGLG1, glycogenin, which is the essential enzyme for the de novo starch synthesis and is regulated by TOR signaling in *C. merolae* (Pancha et al. 2019). In this study, CmGWD is also regulated by TOR signaling, and its dephosphorylation resulted in starch accumulation. Specifically, these results indicate that starch amount will be enhanced by upregulating starch synthesis with activated CmGLG1 and downregulating starch degradation with inactivated CmGWD at the same time through TOR inhibition (Fig. 7).

Consistent with the function of CmGWD (Fig. 3), the reduction of starch in the dark was alleviated in the CmGWD knockout strain (Fig. 4). Interestingly, under conditions where the control strain was exposed to light following dark adaptation, a significant increase in starch (1.30-fold) was observed, whereas in the CmGWD knockout strain, there was a negligible induction (1.05-fold). This discrepancy was further pronounced under conditions supplemented with rapamycin (2.02- and 1.19-fold, respectively), implying possibility of inhibitory control mechanisms to maintain starch homeostasis since the CmGWD knockout strain accumulates a higher basal level of starch. In green algae and land plants, it is well-documented that excess carbon is initially stored as starch before being converted to TAGs (Fan et al. 2012), which may support this hypothesis. The additional increase in starch levels following rapamycin treatment may be attributed to enhanced dephosphorylation of CmGLG1 (the active form), coupled with the inactivation of CmGWD through increased dephosphorylation of Ser264. This suggests that CmGWD and CmGLG1 may serve as potential targets for manipulating starch accumulation in *C. merolae*.

In the CmGWD knockout strain, the amount of phosphorylation per starch was reduced to 0.6-fold that of the control strain, even though *C. merolae* does not possess PWD (Fig. 3C). However, in the case of Arabidopsis, the removal of GWD results in the loss of almost all starch phosphate (Yu et al. 2001). This raises the possibility that there is another starch phosphorylating enzyme or mechanism in *C. merolae*. In our previous study, LC-MS/MS analysis indicated that the phosphorylation status of 5 protein kinases changed after rapamycin treatment (Pancha et al. 2019). One possibility is that these protein kinases are involved in starch phosphorylation. Investigating this hypothesis, along with exploring other potential mechanisms, will be critical for understanding the underlying molecular mechanisms of starch metabolism.

Since TOR and GWD are widely conserved in eukaryotes, the mechanism by which TOR regulates starch accumulation by regulating GWD activity may be widely conserved in eukaryotes. To check this, we analyzed the amino acid sequence of residue Ser264 of GWD and corresponding proteins in other red algae, including *Cyanidiococcus yangmingshanensis* (Liu et al. 2020). Although we could not identify any serine or threonine residue corresponding to CmGWD Ser264 in published red algal GWD, Ser264 residue is conserved in several crop plants such as rice, barley, and wheat (Supplementary Fig. S5). This indicates conservation of such regulation as well as important aspects to explore such mechanisms for improving crop starch production. Improving the crop yield, particularly starch production, is a very important issue in current global food crisis scenarios. Additionally, our analysis indicates that SP sites recognized by proline-directed kinases, including TOR, were found in all red algal GWD proteins analyzed as well as in the GWD of Chlamydomonas and Arabidopsis. All these findings support the possibility that the regulation of starch accumulation via the phosphorylation status of GWD through the TOR signaling pathway is conserved in photosynthetic eukaryotes in general. As mentioned in the introduction, unlike green algae and land plants that accumulate the starch in the chloroplasts, red algae accumulate starch in the cytoplasm, which is called floridean starch. Its properties slightly differ from those of the starch of green algae and land plants in that it contains a higher amount of amylose than amylopectin (Hirabaru et al. 2010). However, regulators involved in starch accumulation in green algae and land plants, such as SEX4 and LSF2, and different group of amylases are also conserved in red algae (Barbier et al. 2005). On the other hand, the domain involved in the redox regulation of GWD was not conserved in red algae (Fig. 3A). These findings suggest a mixture of eukaryotic common and red algal-specific regulations are involved in starch metabolism in red algae. From an evolutionary perspective, previous studies have suggested that starch metabolism itself arose from the coalescence of cyanobacterial and eukaryotic pathways of storage polysaccharide metabolism after cyanobacterial endosymbiosis. In addition, the reconstruction of starch metabolism in the common ancestor of archaea suggests that polysaccharide synthesis was ancestrally cytoplasmic (Ball et al. 2011). Thus, the regulation of starch accumulation by TOR via CmGWD and CmGLG1 (Pancha et al. 2019) elucidated in *C. merolae* can be considered to colorfully reflect a mechanism of control after the birth of photosynthetic eukaryotes.

Analysis of the secondary structure of CmGWD indicates that the N-terminal contains CBM45 module (Fig. 2B). CBM45 is a noncatalytic starch binding module of the GWD, which helps in the binding of GWD to the starch. Generally, the semicrystalline structure of the starch is difficult to hydrolyze by hydrolyzing enzymes, but CBM will help in hydrolyzing this structure (Glaring et al. 2001). Ser264 of CmGWD is located in the first CBM45 module, which is mainly responsible for starch metabolism (Lorberth et al. 1998). These suggest that the phosphorylation of Ser264 of CmGWD may modulate the CmGWD function by changing the CBM45 role.

*A. thaliana* GWD has been reported to show strong changes in transcript abundance but no changes in protein abundance during day/night cycles (Skeffington et al. 2014). Previous studies have also indicated that starch amounts were not changed by GWD overexpression in land plants such as *A. thaliana* and *Hordeum vulgare* (Carciofi et al. 2011; Skeffington et al. 2014). Those findings indicate that the activity of *A. thaliana* GWD is mainly regulated by posttranslational modification including the redox regulation (Mikkelsen et al. 2005). On the other hand, Skeffington et al. found no evidence that the redox regulation of GWD is important for regulating starch degradation in leaves during the night in Arabidopsis (Skeffington et al. 2014). Transgenic *A. thaliana* expressing redox-insensitive GWD, which lacks the Cys residue in the redox regulatory element (Fig. 3A), normally initiates starch degradation at the beginning of the night, with starch degradation rates similar to those of wild-type plants (Skeffington et al. 2014). Thus, it is suggested that redox regulation of GWD is not fully required for regulating the initiation of starch degradation. In this study, it was demonstrated that the CmGWD function is regulated by its phosphorylation status, raising the possibility that phosphorylation of GWD by the TOR signaling pathway is the primary mechanism for GWD regulation in plant lineages like *C. merolae*. However, we still do not have clear data on whether TOR directly regulates GWD activity through phosphorylation or indirectly through another kinase (Fig. 7). A future study on such aspects might be an interesting approach to understanding this mechanism. Another possibility is the existence of regulatory factors that function in correlation with the phosphorylation state of CmGWD, and the results of this study may provide clues to further elucidate the molecular mechanisms of starch accumulation and degradation in plant lineages. Since starch accumulation by TOR inactivation is common in photosynthetic organisms that synthesize and degrade starch in chloroplasts, regulation of phosphorylation by the TOR pathway may also be important in controlling GWD activity in chloroplasts. This point will be clarified by elucidating the regulatory mechanism of GWD by a TOR-signaling pathway other than red algae, which is an issue to be resolved in the future. These results will not only provide important information for the elucidation and evolution of the regulatory mechanisms of starch degradation in plant lineages but will also be utilized in producing biofuels and industrially relevant compounds from starch.

## Materials and methods

### Strain and growth conditions

The *C. merolae* SF12 strain (Imamura et al. 2017) was used for the present study and was grown in an MA2 medium (Imamura et al. 2010). The strain grown at 40 °C under continuous white light (50 *µ*mol m^−2^sec^−1^) with 2% CO_2_ (v/v) bubbling. For the TOR inactivation condition, *C. merolae* SF12 cells (OD_750_ = 0.2 to 0.4) were treated with 2 *µ*M rapamycin at final concentration. For the control experiments, the same amount of DMSO (solvent of rapamycin) was added into the culture medium (Imamura et al. 2018). In the case of nitrate growth conditions, each *C. merolae* strain was grown the same as reported previously (Imamura et al. 2010).

### Phylogenetic analysis

A phylogenetic tree based on 927 unambiguously aligned amino acid positions of GWD and PWD proteins and *C. merolae* CmGWD was constructed as described previously (Imamura et al. 2003).

### Starch measurement in *C. merolae* cells

For starch measurement, 1 mL of *C. merolae* culture was centrifuged at 12,000 g for 10 min, the supernatant was discarded, and starch content was measured from the pellets (Takusagawa et al. 2016). In detail, 100 *µ*L of 10% (w/v) sodium dodecyl sulfate (SDS) was added to the pellets and incubated at room temperature for 15 min. After incubation, the complete solution was centrifuged at 12,000 g for 10 min, supernatant was discarded, and pellets were washed with 50 °C prewarmed 80% (v/v) ethanol. The solution was vortexed for 5 min. After vortexing, the solution was centrifuged at 12,000 g for 10 min, and the pellets were dissolved in 75 *µ*L milliQ water and incubated at 95 °C for 15 min. After incubation, the solution was cooled at room temperature, and then, 125 *µ*L of 60% perchloric acid (FUJIFILM Wako Pure Chemical Corporation, Osaka, Japan) was added and vortexed for 15 min. After vortexing, 300 *µ*L of milliQ water was added to the solution and mixed well. After centrifugation at 12,000 g for 10 min, the resulting supernatant was used to measure the sugar content using phenol-sulfuric acid method. In detail, 200 *µ*L of 5% (w/v) phenol solution was added to 200 *µ*l of extracted sugar from *C. merolae* cells. One milliliter of concentrated sulfuric acid was added to it. The solution was incubated at 40 °C for 20 min, and the absorbance was determined at 490 nm (Dubois et al. 1956). A standard curve was prepared using 20 to 200 *µ*g/200 *µ*L glucose solutions. The sugar content was converted to pg cell^−1^.

### Quantification of starch phosphate in *C. merolae* cells

To determine the phosphate content in starch, 1 mL of *C. merolae* cells was transferred to a micro-centrifugation tube and 2 *µ*L of 0.5% (v/v) Tween 20 was added to the tube and centrifuged at 10,000 g for 10 min. Twenty percent (w/v) SDS was added to the resultant pellets and incubated at 15 min at room temperature. After incubation, the solution was centrifuged at 10,000 g for 10 min. The pellets were suspended in 50 °C prewarmed ethanol and vortexed for 5 min. The solution was again centrifuged at 10,000 g for 10 min. To the pellets, 20 *µ*L of concentrated sulfuric acid was added and allowed to stand for 24 h. After incubation, the solution was neutralized with 360 *µ*L of 1 m potassium hydroxide solution. Phosphoric acid content of the solution was determined using PiBlue Phosphate assay kit (BioAssay System, CA, USA).

### Immunoblot analysis

For immunoblot analysis, approximately 20 mL *C. merolae* cells (OD_750_ = 0.2 to 0.4) was centrifuged at 12,000 g for 10 min. The resulting pellets were dissolved in 250 *µ*L of extraction buffer (25 mm Tris-HCl pH 8.0, OComplete Mini EDTA free protease inhibitor and phosphatase inhibitor cocktail). One hundred milligrams of glass beads (≤106 *µ*m) was added to the pellets and vortexed at 4 °C (scale 8, 5 min, TurboMix attached to Vortex Genie 2; Scientific Industries, New York, USA). After vortexing, the solution was centrifuged at 12,000 g for 10 min, and supernatant was used for immunoblot analysis. The protein solution was mixed with an equal volume of 2× sample buffer (Laemmli 1970) and incubated at 37 °C for 30 min before SDS–polyacrylamide gel electrophoresis (PAGE). FLAG-fused protein was detected using antibodies against FLAG-tag (Thermo Fisher Scientific, Tokyo, Japan).

### Phos-tag agarose and immunoblot analyses

To determine the phosphorylation status of CmGWD, Phos-tag agarose analysis was performed as described previously (Kinoshita-Kikuta et al. 2009). In the first step, a spin centrifuge tube was prepared using a 0.5 mL PCR tube. A small hole was made at the bottom of the tube using 21-G needle (TERUMO, Tokyo, Japan). A Phos-tag agarose (Thermo Fisher Scientific, Tokyo, Japan) was transferred to a prepared spin centrifuge tube. The tube was centrifuged at 2,000 g for 20 s to remove the storage buffer. Forty microliters of balancing buffer (0.1 m Tris-CH_3_COOH pH 7.5, 1.0 m CH_3_COONa and 10 *µ*M Zn(OCOCH_3_)_2_) was added and incubated in the tube for 5 min at room temperature. The tube was centrifuged at 2,000 g for 20 s, and the resulting filtrate was discarded. The agarose beads were then washed with 40 *µ*L of binding/washing solution (0.1 m Tris-CH_3_COOH pH 7.5, 1.0 m CH_3_COONa). Finally, 15 *µ*g total protein was applied to the washed agarose beads and incubated for 5 min at room temperature. The whole unit was centrifuged at 2,000 g for 20 s. The flow-through fraction was collected as dephosphorylated proteins. The agarose beads were washed once using 100 *µ*L binding/washing solution. Distilled water (40 *µ*L or more) was applied to collect agarose beads in a new centrifuge tube. The tube was centrifuged at 2,000 g for 20 s, and supernatant was removed. The agarose beads contained phosphorylated fractions of the protein. The total protein, flow-through fraction (dephosphorylated proteins), and collected agarose beads (phosphorylated proteins) were mixed with an equal volume of 2× sample buffer (Laemmli 1970) and incubated at 37 °C for 30 min. The FLAG-fused protein was detected by an immunoblot analysis using antibodies against FLAG-tag (Thermo Fisher Scientific, Tokyo, Japan).

### Construction of CmGWDox, ΔCmGWD, CmGWD^S264^Aox, CmGWD^S264^Dox, and CmGWD complementation strains

To construct the ΔCmGWD strain, the ORF region of the genome was disrupted by homologous recombination by inserting an additional DNA fragment (approx. 1,500 bp) to the upstream and downstream of CMT547C ORF to both ends of the selection marker URA5.3T (Takemura et al. 2018). To construct the DNA fragments, CMT547C upstream (−2,500 to −1,000, with respect to the CMT547C ORF initiation site) and downstream (+1,516 to +3,016) regions were PCR-amplified using *C. merolae* genomic DNA as a template and the specific primer sets F1 (5′-GACACGTGAATTTAAATAGGCGGACCCGTTGCACTGCACC-3′), R1(5′-TGAAAATAAAGATTTTCAGGACTCGTTGGCTCTGTGGAAT-3′), F2 (5′-AGGGATTGTGGCGCGACAGGTAGATATCGGTCGTTGAATG-3′), and R2 (5′-GACACGTGAATTTAAATAGGTTCCAATGCACGCGTCAAGG-3′), respectively. The PCR-amplified fragment was cloned into the *Stu*I digested pMKTf plasmid (Takemura et al. 2018) using Gibson assembly Master Mix (New England Biolabs, Tokyo, Japan) to create pMKT-T547. To construct pCmGWDox plasmid, the CmGWD (CMT547C) gene was amplified using primers F3 (5′-TTTCTTCGTTCGTTGACCCCCATGAGCGATCAACCAAGCG-3′) and R3 (5′-TGCAGGTCGACTCTAGACCCGACTTGGTCACGTGATTGAA-3′) using *C. merolae* genomic DNA as templet. pSUGA contains a strong *APCC* promoter and FLAG epitope tag coding sequence as expression vector (Fujii et al. 2013). The amplified fragment was cloned into *Sma*I-digested pSUGA using a SLiCE method (Zhang et al. 2012) to construct pGWD. To construct a pGWD^S264A^ox plasmid, a joint PCR method was employed. Fragment 1 was amplified using primers F4 (5′-GTTTCACGCAGTGATGAGTACATGTCGCTTTCACGGGAAA-3′) and R3 (5′-TGCAGGTCGACTCTAGACCCGACTTGGTCACGTGATTGAA-3′) using *C. merolae* genomic DNA as template. In contrast, Fragment 2 was amplified using F3 (5′-TTTCTTCGTTCGTTGACCCCCATGAGCGATCAACCAAGCG-3′) and R4 (5′-TGAAAGCGACATGTACTCATCACTGCGTGAAACACCTAATGCCGTC-3′) using *C. merolae* genomic DNA as template. After obtaining both fragments, a joint PCR was conducted to construct a pGWD^S264A^ fragment. Similarly, to construct a pGWD^S264D^ plasmid, fragments 1 and 2 were constructed using primers F5 (5′-GTTTCACGCAGTGATGCGTACATGTCGCTTTCACGGGAAA-3′) and R3 (5′-TGCAGGTCGACTCTAGACCCGACTTGGTCACGTGATTGAA-3′) and F3 (5′-TTTCTTCGTTCGTTGACCCCCATGAGCGATCAACCAAGCG-3′) and R5 (5′-TGAAAGCGACATGTACGCATCACTGCGTGAAACACCTAATGCCGTC-3′) for using *C. merolae* genomic DNA as templet. After a joint fragment was obtained, it was cloned into the *Sma*I-digested pSUGA using a SLiCE method (Zhang et al. 2012) to construct the pGWD^S264A^ and pGWD^S264D^ plasmids. In the case of inducting CmGWD proteins by nitrate growth conditions, 3 plasmids were constructed: pNITE-GDW, pNITE-GWD^S264A^, and pNITE-GWD^S264D^. For each pNITE-based plasmid, PCR-amplified fragments were obtained using a primer set: T547 pNITE forward (5′-TACTCTGCCCGGGTCTAGAGATGAGCGATCAACCAAGCGT-3′) and T547 pNITE reverse (5′-TCGCATGCCTGCAGGTCGACGACTTGGTCACGTGATTGAA-3′) with *C. merolae* genomic DNA as a template. The resultant fragments were cloned into a *Xba*I-digested pNITE using a SLiCE method to construct pNITE-GDW, pNITE-GWD^S264A^, and pNITE-GWD^S264D^. The obtained plasmids were transformed into the SF12 host strain as described previously (Imamura et al. 2017). After incubation for 2 to 3 weeks, the resulting colonies were confirmed by colony PCR using primers F6 (5′-ATGGAGCCCCAGTCCGAGTA-3′) and R6 (5′-TCACTTGTCATCGTCATCCTTGTAATCGAT-3′). Further, the expression of FLAG-fused proteins (CmGWD, CmGWD^S264A^, and CmGWD^S264D^) was confirmed by immunoblot analysis. A control strain was also obtained by transforming SF12 with the empty vector pSUGA. To obtain the CmGWD complementation strain, approximately 1 × 10^8^ ΔCmGWD (uracil autotrophy) strain cells were spread on an MA2 Gellan Gum plate containing 0.5 mg/mL uracil and 0.8 mg/mL 5-FOA (Takemura et al. 2019a, 2019b). The plate was incubated in a bag with AnaeroPack CO_2_ (Mitsubishi Gas Chemical Co., Inc., Tokyo, Japan) for 4 weeks, and colonies were picked and transferred into 2 mL of MA2 medium supplemented with 0.5 mg/mL uracil and 0.8 mg/mL 5-FOA for an additional 2 weeks. After confirming the removal of the marker, *URA5.3T*, by PCR as previously reported (Imamura et al. 2017), the strain was used as a host for transformation with pCmGWDox to obtain the CmGWD complementation strain.

### Accession numbers

Sequence data from this article can be found in the GenBank/EMBL data libraries under the following accession numbers: CmGWD, XP_005539500; CmGLG1, XP_005536455.

## Supplementary Material

kiaf106_Supplementary_Data

## Data Availability

All data generated or analyzed during this study are included in this published article and its online supplementary data. Information on *C. merolae* genes, such as sequence and description, is based on the *Cyanidioschyzon merolae* database (http://czon.jp/).

## Supplementary data

The following materials are available in the online version of this article.

**Supplementary Figure S1.** LC-MS/MS identified phosphorylation at S264 and S291 of CmGWD.

**Supplementary Figure S2.** CmGWD, P-CmGWD, and DeP-CmGWD protein levels after rapamycin treatment.

**Supplementary Figure S3.** Confirmation of CmGWD knockout strain, ΔCmGWD.

**Supplementary Figure S4.** Confirmation of CmGWD complementation in the ΔCmGWD.

**Supplementary Figure S5.** Conservation of Ser264 residue.

## Dive Curated Terms

The following phenotypic, genotypic, and functional terms are of significance to the work described in this paper:

BAM3 Gramene: AT4G20270
BAM3 Araport: AT4G20270

ISA3 Gramene: AT4G09020
ISA3 Araport: AT4G09020

starch CHEBI: CHEBI:28017

PWD Gramene: AT5G26570
PWD Araport: AT5G26570

BAM1 Gramene: AT3G23920
BAM1 Araport: AT3G23920

AMY3 Gramene: AT1G69830
AMY3 Araport: AT1G69830
