# Supplementary material for: Target of rapamycin signaling regulates starch degradation via α-glucan water dikinase in a unicellular red alga
Source: Plant Physiol. 2025 Mar 21;197(4):kiaf106. doi: 10.1093/plphys/kiaf106 (PMC11986951; doi:10.1093/plphys/kiaf106)
Supplement: kiaf106_Supplementary_Data [file kiaf106_supplementary_data.zip › Figs.S1-S5.pdf]

Supplementary Figure S1

(a)

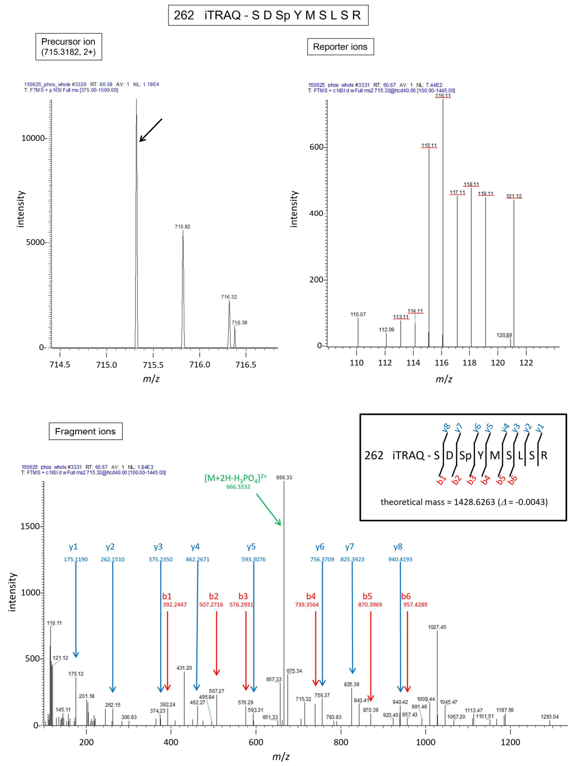

(b)

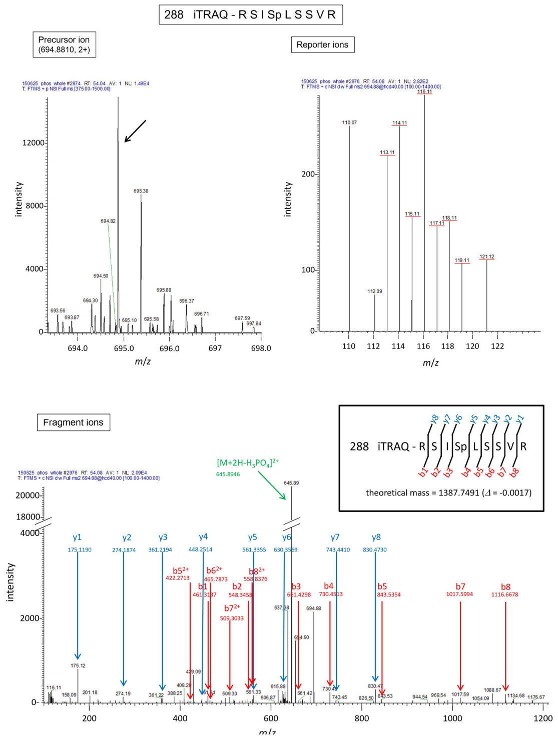

(c)

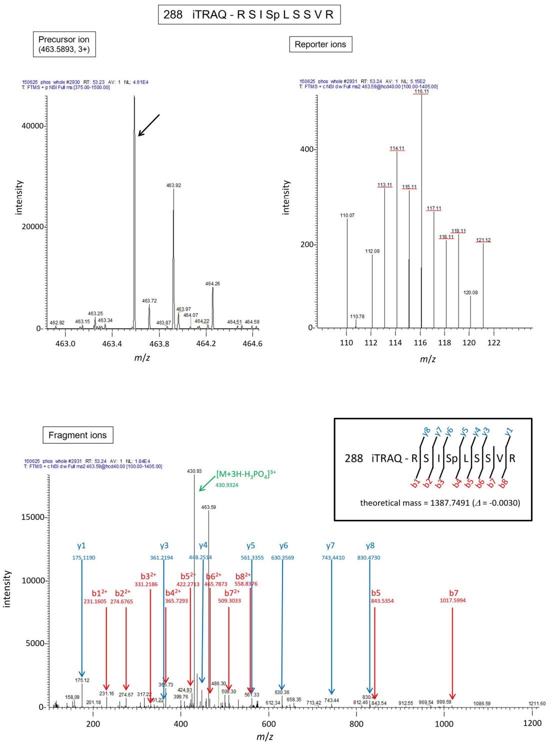

Supplementary Figure S2

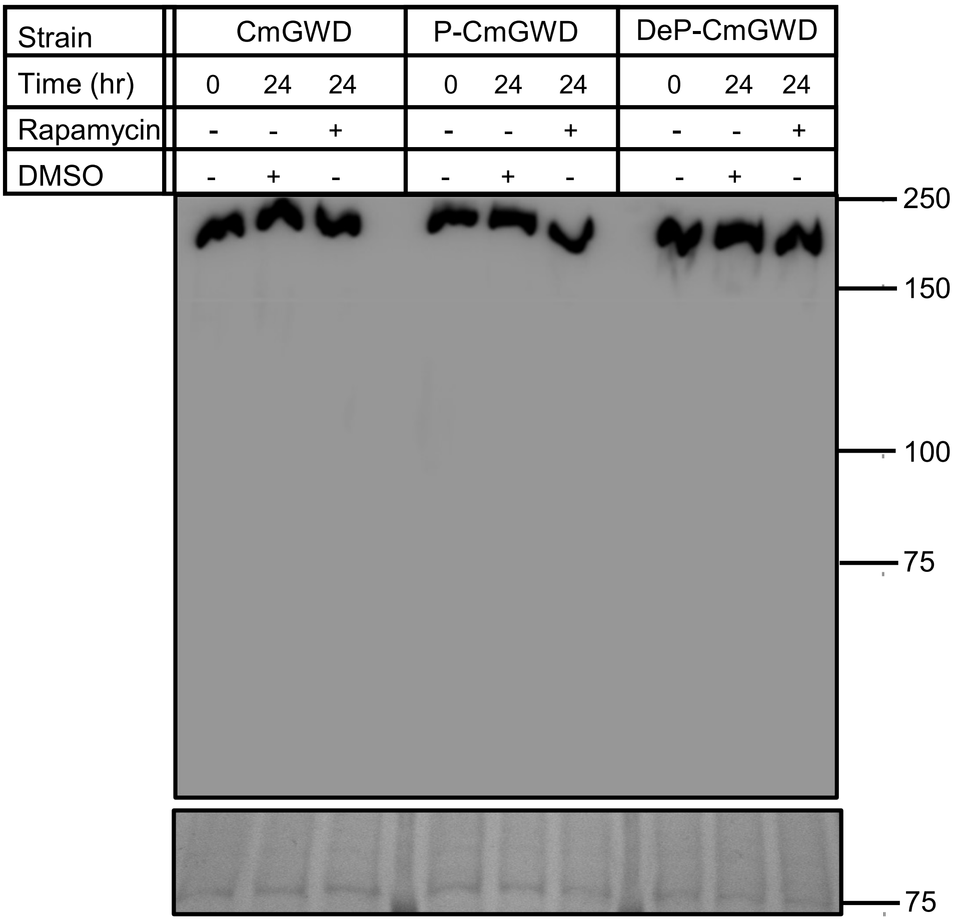

Supplementary Figure S3

(a)

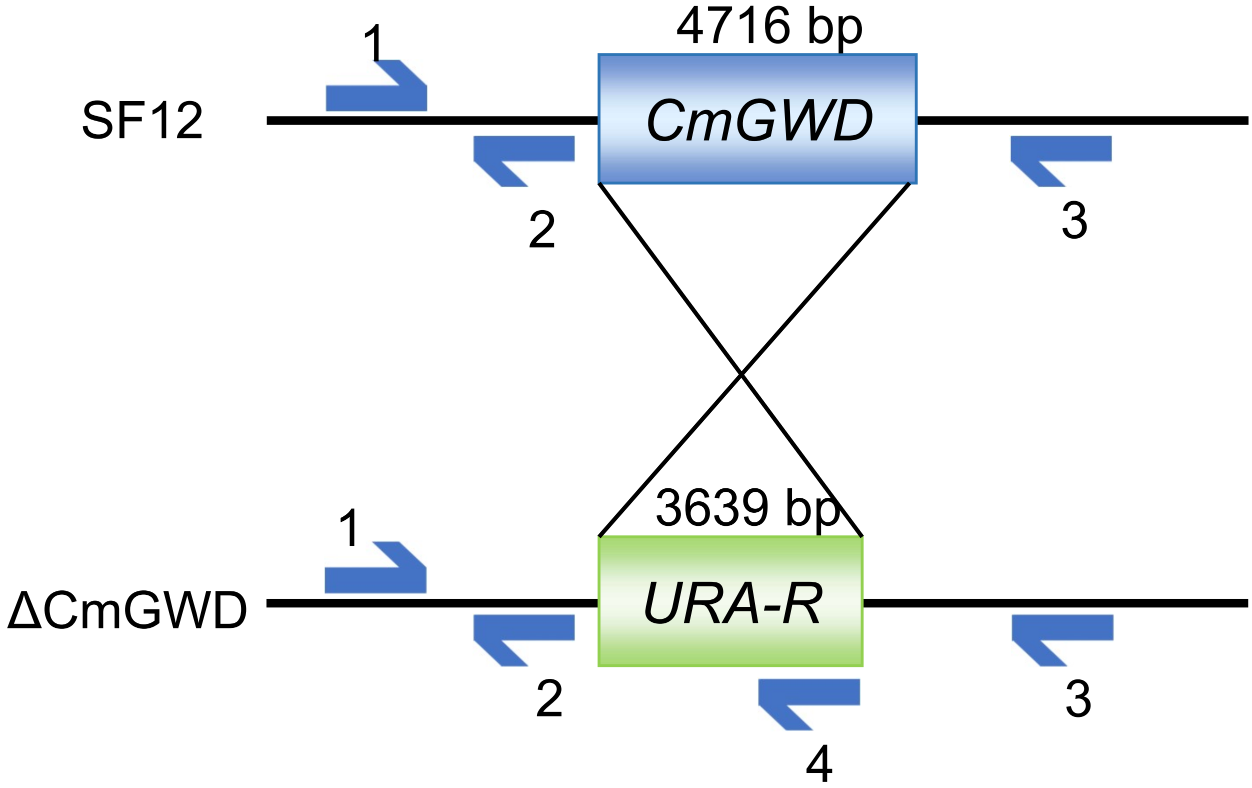

(b)

Pairs of primer set	1 and 3		1 and 2		1 and 4		
Predicted PCR product size	6456	5379	1020	1020	-	4653	(bp)
Strains	SF12	ΔCmGWD	SF12	ΔCmGWD	SF12	ΔCmGWD	Marker

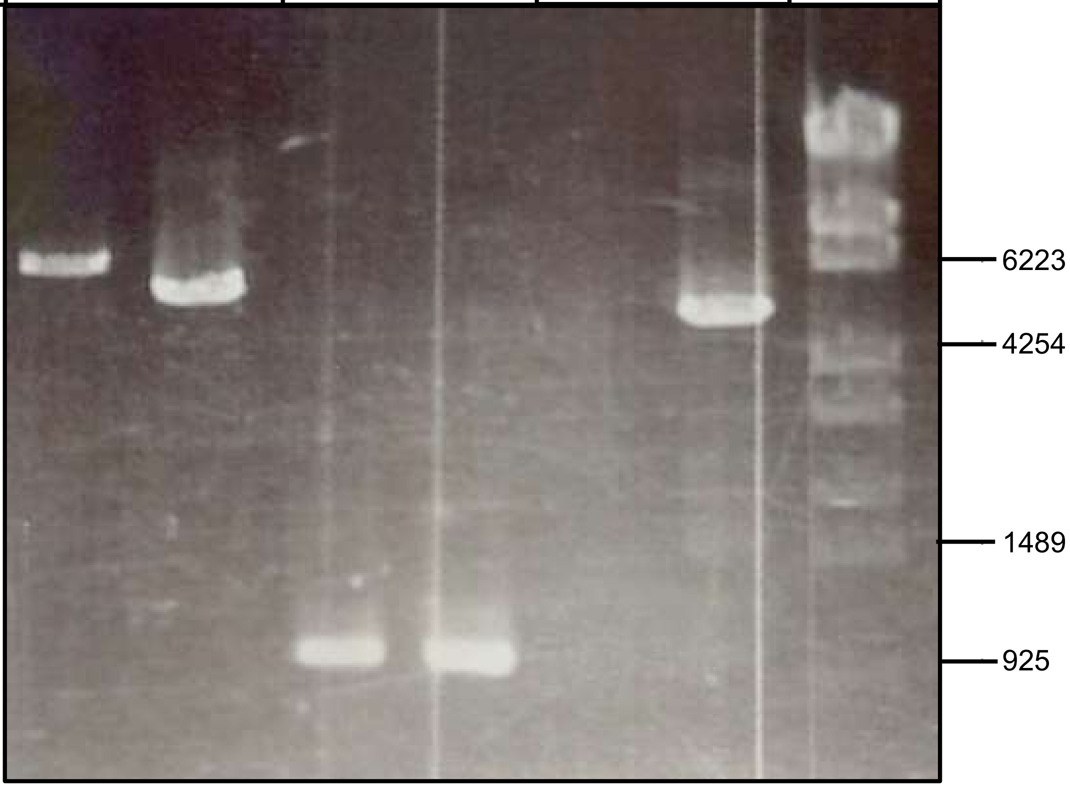

Supplementary Figure S4

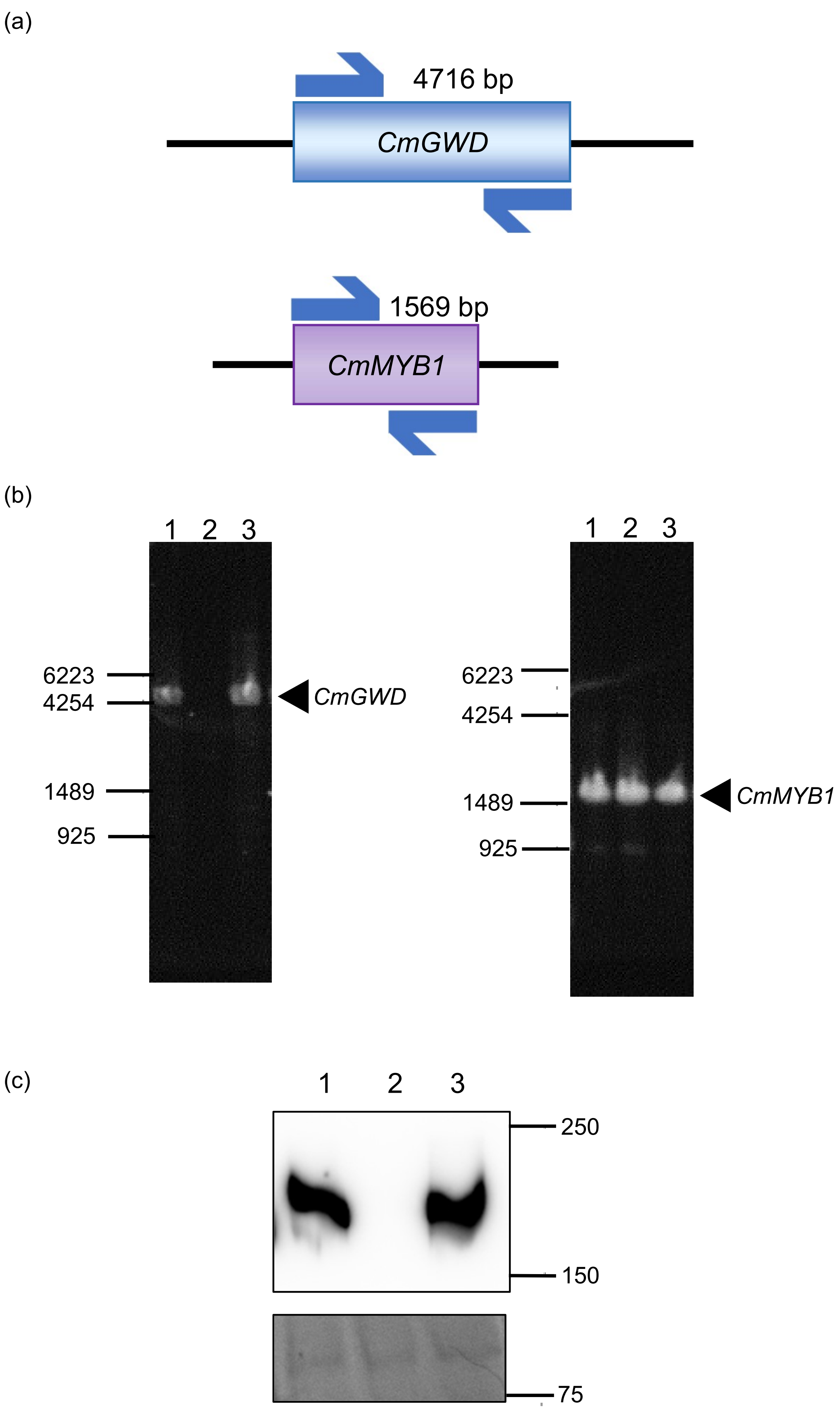

Supplementary Figure S5

Conserved
Serine residue

	* (conserved serine)
*C. merolae*_GWD	VSRSDSYMSLSRETSPG-PDA
*S. tuberosum*_GWD	LVQIQSYLRWERKGKQNYTPE
*S. chacoense*_GWD	LVQIQSYLRWERKGKQNYTPE
*T. aestivum*_GWD	LVQIQSYLRWERKGNQNYTPE
*H. vulgare*_GWD	LVQIQSYLRWERKGNQNYTPE
*B. distachyon*_GWD	LVQIQSYLRWERKGNQNYTPE
*O. sativa*_GWD	LVQIQSYLRWERKGKQNYTPE
*P. patens*_GWD	LVGIQSYLRWERMGRQNYSPE
