# Supplementary material for: Target of rapamycin signaling regulates starch degradation via α-glucan water dikinase in a unicellular red alga
Source: Plant Physiol. 2025 Mar 21;197(4):kiaf106. doi: 10.1093/plphys/kiaf106 (PMC11986951; doi:10.1093/plphys/kiaf106)
Supplement: kiaf106_Supplementary_Data [file kiaf106_supplementary_data.zip › Supporting Text20241122-final.docx]

**Supporting Text**

**Supporting Figure S1. LC-MS/MS identified phosphorylation at S264 and S291 of the CmGWD protein.**

**a**, The MS/MS spectra that led to identifying peptide 262-270 with phosphorylation at S264 are shown: the precursor ion (upper left), b and y fragment ions with their theoretical masses (lower) and iTRAQ reporter ions (upper right). The *P*-value for the identification given by the MASCOT search engine was 2.3×10-4. Note that the search engine also suggested phosphorylation at S262 with a significant but larger *P*-value (2.9×10-3) based on the same spectra. **b** and **c**, The MS/MS spectra that led to identifying peptide 288-296 with phosphorylation at S291 are shown. The *P*-values were 1.2×10-3 (panel b) and 3.5×10-3 (panel c).

**Supporting Figure S2. CmGWD, P-CmGWD, DeP-CmGWD protein levels after rapamycin treatment.**

Aliquots of total protein (8.0 µg each) isolated from each indicated strain under the specified conditions were separated by 7% sodium dodecyl sulfate-polyacrylamide gel electrophoresis (SDS-PAGE) and analyzed by immunoblotting with a monoclonal anti-FLAG antibody. Molecular size marker positions are indicated in kDa on the right. After antibody detection of a signal, the membrane was stained with Coomassie Brilliant Blue, which was used as a loading control (lower panel).

**Supporting Figure S3. Confirmation of CmGWD knockout strain, ΔCmGWD.**

**a**, Schematic diagram for construction of ΔCmGWD. SF12 and ΔCmGWD indicate *C. merolae* SF12 and CmGWD knockout strain. *URA-R* is the uracil synthetase, a marker cassette, for the selection. Arrows with numbers show the primer used in panel b. **b**, Confirmation of the transformation. DNAs extracted from *C. merolae* SF12 strain and ΔCmGWD *C. merolae* strain were analyzed by PCR with a set of primers indicated in the figure. The PCR products were resolved by 1.0% agarose gel electrophoresis. The positions of a molecular size marker are indicated as bp at the right.

**Supporting Figure S4. Confirmation of CmGWD complementation in the ΔCmGWD.**

**a**, Schematic diagram of the position of primers used for PCR. Arrows indicate the primers used in panel b. The numbers with “bp” represent the predicted PCR product sizes. *CmMYB1* was used as a control gene. **b**, Confirmation of the transformation. DNAs were extracted from the *C. merolae* SF12 strain (lane 1), ΔCmGWD strain (lane 2), and the CmGWD complementation strain in the ΔCmGWD background (lane 3). Each strain was analyzed by PCR with a set of primers indicated in panel a. The PCR products were resolved by 1.0% agarose gel electrophoresis. The positions of a molecular size marker are indicated as bp on the left. **c**, Immunoblot analysis was performed the same as in Fig. S2. Lane 1: FLAG-fused CmGWD expressing strain; lane 2: ΔCmGWD; lane 3: CmGWD complementation strain in the ΔCmGWD background.

**Supporting Figure S5. Conservation of Ser264 residue.**

Partial amino acid sequences of *Cyanidioschyzon merolae* GWD, *Solanum tuberosum* GWD, *Solanum chacoense* GWD, *Triticum aestivum* GWD, *Hordeum vulgare* GWD, *Brachypodium distachyon* GWD, *Oryza sativa* GWD, and *Physcomitrella patens* GWD proteins are aligned. Each accession number is XP_005539500.1, AFH88388.1, XP_049351140.1, XP_044426603.1, XP_044956847.1, XP_010227681.1, XP_015643466.1, and XP_024383094.1 as this order, respectively.
